# Refining transcriptional regulatory networks using network evolutionary models and gene histories

**DOI:** 10.1186/1748-7188-5-1

**Published:** 2010-01-04

**Authors:** Xiuwei Zhang, Bernard ME Moret

**Affiliations:** 1 Laboratory for Computational Biology and Bioinformatics, EPFL (Ecole Polytechnique Fédérale de Lausanne), EPFL-IC-LCBB, INJ230, Station 14, CH-1015 Lausanne, Switzerland

## Abstract

**Background:**

Computational inference of transcriptional regulatory networks remains a challenging problem, in part due to the lack of strong network models. In this paper we present evolutionary approaches to improve the inference of regulatory networks for a family of organisms by developing an evolutionary model for these networks and taking advantage of established phylogenetic relationships among these organisms. In previous work, we used a simple evolutionary model and provided extensive simulation results showing that phylogenetic information, combined with such a model, could be used to gain significant improvements on the performance of current inference algorithms.

**Results:**

In this paper, we extend the evolutionary model so as to take into account gene duplications and losses, which are viewed as major drivers in the evolution of regulatory networks. We show how to adapt our evolutionary approach to this new model and provide detailed simulation results, which show significant improvement on the reference network inference algorithms. Different evolutionary histories for gene duplications and losses are studied, showing that our adapted approach is feasible under a broad range of conditions. We also provide results on biological data (*cis*-regulatory modules for 12 species of *Drosophila*), confirming our simulation results.

## Introduction

Transcriptional regulatory networks are models of the cellular regulatory system that governs transcription. Because establishing the topology of the network from bench experiments is very difficult and time-consuming, regulatory networks are commonly inferred from gene-expression data. Various computational models, such as Boolean networks [1], Bayesian networks [2], dynamic Bayesian networks (DBNs) [3], and differential equations [4,5], have been proposed for this purpose, along with associated inference algorithms. Results, however, have proved mixed: the high noise level in the data, the paucity of well studied networks, and the many simplifications made in the models all combine to make inference difficult.

Bioinformatics has long used comparative and, more generally, evolutionary approaches to improve the accuracy of computational analyses. Work by Babu's group [6-8] on the evolution of regulatory networks in *E. coli *and *S. cerevisiae *has demonstrated the applicability of such approaches to regulatory networks. They posit a simple evolutionary model for regulatory networks, under which network edges are simply added or removed; they study how well such a model accounts for the dynamic evolution of the two most studied regulatory networks; they then investigate the evolution of regulatory networks with gene duplications [8], concluding that gene duplication plays a major role, in agreement with other work [9].

Phylogenetic relationships are well established for many groups of organisms; as the regulatory networks evolved along the same lineages, the phylogenetic relationships informed this evolution and so can be used to improve the inference of regulatory networks. Indeed, Bourque and Sankoff [10] developed an integrated algorithm to infer regulatory networks across a group of species whose phylogenetic relationships are known; they used the phylogeny to reconstruct networks under a simple parsimony criterion. In previous work [11], we presented two refinement algorithms, both based on phylogenetic information and using a likelihood framework, that boost the performance of any chosen network inference method. On simulated data, the *receiver-operator characteristic (ROC) *curves for our algorithms consistently dominated those of the standard approaches used alone; under comparable conditions, they also dominated the results from Bourque and Sankoff. Both our previous approach and that of Bourque and Sankoff are based on an evolutionary model that considers only edge gains and losses, so that the networks must all have the same number of genes (orthologous across all species). Moreover, the gain or loss of an edge in that model is independent of any other event. However, this process accounts for only a small part of regulatory network evolution; in particular, gene duplication is known to be a crucial source of new genetic function and a mechanism of evolutionary novelty [8,9].

In this paper we present a model of network evolution that takes into account gene duplications and losses and their effect on regulatory network structures. Such a model provides a direct evolutionary mechanism for edge gains and losses, while also enabling broader application and more flexible parameterization. For example, in the networks to be refined, the genes can have different numbers of copies for different organisms. Within this broader framework, the phylogenetic information that we use lies on two levels: the evolution of gene contents of the networks and the regulatory interactions of the networks. The former can be regarded as the basis of the latter, and can be obtained by inferring the history of gene duplications and losses during evolution. We then extend our refinement algorithms [11] to handle this data and use different models of gene duplications and losses to study their effect on the performance of the refinement algorithms.

Our experimental results confirm that our new algorithms provide significant improvements over the base inference algorithms, and support our analysis of the performance of refinement algorithms under different models of gene duplications and losses.

## Background

Our refinement algorithms [11] work iteratively in two phases after an initialization step. First, we obtain the regulatory networks for the family of organisms; typically, these networks are inferred from gene-expression data for these organisms, using standard inference methods. We place these networks at the corresponding leaves of the phylogeny of the family of organisms and encode them into binary strings by simply concatenating the rows of their adjacency matrix. We then enter the iterative refinement cycle. In the first phase, we infer ancestral networks for the phylogeny (strings labelling internal nodes), using our own adaptation of the FastML[12] algorithm; in the second phase, these ancestral networks are used to refine the leaf networks. These two phases are then repeated as needed. Our refinement algorithms are formulated within a maximum likelihood (ML) framework and focused solely on refinement--they are algorithmic boosters for one's preferred network inference method. Our new algorithms retain the same general approach, but include many changes to use the duplication/loss data and handle the new model.

### Base network inference methods

We use both DBN and differential equation models as base inference methods in our experiments. When DBNs are used to model regulatory networks, an associated structure-learning algorithm is used to infer the networks from gene-expression data [3,13,14]; so as to avoid overly complex networks, a penalty on graph structure complexity is usually added to the ML score, thereby reducing the number of false positive edges. In [11] we used a coefficient *k*_*p *_to adjust the weight of this penalty and studied different tradeoffs between sensitivity and specificity, yielding the optimization criterion log *Pr*(*D*|*G*, ) - *k*_*p*_#*G *log *N*, where *D *denotes the dataset used in learning, *G *is the (structure of the) network,  is the ML estimate of parameters for *G*, #*G *is the number of free parameters of *G*, and *N *is the number of samples in *D*.

In models based on differential equations [4,5], a regulatory network is represented by the equation system *dx*/*dt *= *f*(*x*(*t*))-*Kx*(*t*), where *x*(*t*) = (*x*_1_(*t*),⋯, *x*_*n*_(*t*)) denotes the expression levels of the *n *genes and *K *(a matrix) denotes the degradation rates of the genes. The regulatory relationships among genes are then characterized by *f *(·). To get networks with different levels of sparseness, we applied different thresholds to the connection matrix to get final edges. In our experiments we use Murphy's Bayesian Network Toolbox [15] for the DBN approach and TRNinfer[5] for the differential equation approach; we refer to them as *DBI *and *DEI*, respectively.

### Refinement algorithms in our previous work

The principle of our phylogenetic approach is that phylogenetically close organisms are likely to have similarly close regulatory networks; thus independent network inference errors at the leaves get corrected in the ancestral reconstruction process along the phylogeny. In [11], we gave two refinement algorithms, *RefineFast *and *RefineML*. Each uses the globally optimized parents of the leaves to refine the leaves, but the first simply picks a new leaf network by sampling from the inferred distribution (given the parent network, the evolutionary model parameters, and the phylogeny), while the second combines the inferred distribution with a prior, the existing leaf network, using a precomputed *belief coefficient *that indicates our confidence in the current leaf network, and returns the most likely network under these parameters.

### Inference of gene duplication and loss history

To infer ancestral networks with the extended network evolution model, we need a full history of gene duplications and losses for the gene families. Reconciliation of gene trees with the species tree [16-18] is one way to infer this history. The species tree is the phylogenetic tree whose leaves correspond to the modern organisms; gene duplications and losses occur along the branches of this tree. A gene tree is a phylogenetic tree whose leaves correspond to genes in orthologous gene families across the organisms of interest; in such a tree, gene duplications and speciations are associated with internal nodes.

When gene duplications and losses occur, the species trees and the gene trees may legitimately differ in topology. Reconciling these superficially conflicting topologies--that is, explaining the differences through a history of gene duplications and losses--is known as *lineage sorting *or *reconciliation *and produces a list of gene duplications and losses along each edge in the species tree. While reconciliation is a hard computational problem, algorithms have been devised for it in a Bayesian framework [16] or using a simple parsimony criterion [17]. In our experiments, we use the parsimony-based reconciliation tool Notung [17], but we also investigate the effect of using alternate histories.

## The evolutionary model

### The new network evolutionary model

Although transcriptional regulatory networks produced from bench experiments are available for only a few model organisms, other types of data have been used to assist in the comparative study of regulatory mechanisms across organisms [19-21]. For example, gene-expression data [21], sequence data like transcriptional factor binding site (TFBS) data [19,20], and *cis*-regulatory elements [21] have all been used in this context. Moreover, a broad range of model organisms have been studied, including bacteria [7], yeast [19,21], and fruit fly [20]. While these studies offer some insights, they have not to date sufficed to establish a clear model for regulatory networks or their evolution. Our new model remains simple, but can easily be generalized or associated with other models, such as the evolutionary model of TFBSs [22]. In this new model, the networks are represented by binary adjacency matrices.

The evolutionary operations are:

• *Gene duplication*: a gene is duplicated with probability *p*_*d*_. After a duplication, edges for the newly generated copy can be assigned as follows:

*Neutral initialization: *Create connections between the new copy and other genes randomly according to the proportion π_1 _of edges in the background network independently of the original copy.

*Inheritance initialization: *Connections of the duplicated copy are reported to correlate with those of the original copy [7-9]. This observation suggests letting the new copy inherit the connections of the original, then lose some of them or gain new ones at some fixed rate [23].

*Preferential attachment: *The new copy gets preferentially connected to genes with high connectivity [23,24].

• *Gene loss*: a gene is deleted along with all its connections with probability *p*_*l*_.

• *Edge gain*: an edge between two genes is generated with probability *p*_01_.

• *Edge loss*: an existing edge is deleted with probability *p*_10_.

The model parameters are thus *p*_*d*_, *p*_*l*_, the proportions of 0 s and 1 s in the networks Π = (π_0 _π_1_), the substitution matrix of 0 s and 1 s, , plus parameters suitable to the initialization model.

### Models of gene duplications and losses

While networks evolve according to the network evolutionary model described above, a history of gene duplications and losses is created along the evolution. However, during reconstruction, this history may not be exactly reconstructed. Therefore, we propose other models of gene duplications and losses to approximate the true history:

• *The duplication-only model*: We assume that different gene contents are due exclusively to gene duplication events.

• *The loss-only model*: We assume that different gene contents are due exclusively to gene loss events.

We also compare outcomes when the true history is known.

## The new refinement methods

We begin by collecting the regulatory networks to be refined. These networks may have already been inferred or they can be inferred from gene-expression data at this point using any of the standard network inference methods. The genes in these networks are not required to be orthologous across all species, as the duplication/loss model allows for gene families of various sizes. Refinement proceeds in the two-phase iterative manner already described, but adding a step for reconstruction of gene duplication and loss history and suitably modified algorithms for ancestral reconstruction and leaf refinement:

1. Reconstruct the history of gene duplications and losses, from which the gene contents for the ancestral regulatory networks (at each internal node of the species tree) can be determined. We present algorithms for history reconstruction with different gene duplication and loss models.

2. Infer the edges in the ancestral networks once we have the genes of these networks. We do this using a revised version of FastML.

3. Refine the leaf networks with new versions of *RefineFast *and *RefineML*.

4. Repeat steps 2 and 3 as needed.

### Inferring gene duplication and loss history

The *duplication-only *and *loss-only *models allow simplifying the inference of the gene duplication and loss history and of the gene contents of the ancestors. For a certain internal node of the phylogenetic tree, with the *duplication-only *assumption, the intersection of the genes of all the leaves in the subtree rooted at this internal node is its set of genes, while with the *loss-only *assumption, the union of genes in all the leaves of the subtree is the set of genes. Gene duplication and loss histories inferred with these methods have a minimum number of gene duplications, respectively losses -- they are optimal under the model.

With both operations allowed, there are different ways of getting such a history. For example, the reconciliation algorithms introduced earlier can be used to reconstruct the history. Besides, the orthology assignment of the gene families across species can be leveraged for better inference of the history. FastML[12], which was designed to infer ancestral sequences given the sequences of a family of modern organisms, can be applied in this case after the following preprocessing. Suppose there are *N *different genes in all the modern organisms, we then represent the gene content of each organism with a binary sequence of length *N*, where the value at each position is assigned as 1 if the corresponding gene or its ortholog is present, otherwise 0. FastML can be used to obtain an estimate of these sequences for the ancestral organisms, with a character set {0, 1} and the substitution matrix:

Note that this approach assumes 1-1 orthologies, whereas orthology is a many-to-many relationship. In biological practice, however, 1-1 orthologies are by far the most common.

### Inferring ancestral networks

FastML[12] assumes independence among the entries of the adjacency matrices and reconstructs ancestral characters one site at a time. When the gene content is the same in all networks, FastML can be used nearly unchanged, as in our previous work [11]. In our new model, however, the gene content varies across networks. We solve this problem by embedding all networks into one that includes every gene that appears in any network, taking the union of all gene sets. We then represent a network with a ternary adjacency matrix, where the rows and columns of the missing genes are filled with a special character *x*. All networks are thus represented with adjacency matrices of the same size. Since the gene contents of ancestral networks are known thanks to reconciliation, the entries with *x *are already identified in their matrices; other entries are reconstructed by our revised version of FastML, with a new character set *S' *= {0, 1, *x*}. We modify the substitution matrix and take special measures for *x *during calculation. The substitution matrix *P' *for *S' *can be derived from the model parameters, without introducing new parameters:

Given *P'*, let *i*, *j*, *k *denote a tree node, and *a*, *b*, *c *∈ *S' *possible values of a character at some node. For each character *a *at each node *i*, we maintain two variables:

• *L*_*i*_(*a*): the likelihood of the best reconstruction of the subtree with root *i*, given that the parent of *i *is assigned character *a*.

• *C*_*i*_(*a*): the optimal character for *i*, given that its parent is assigned character *a*.

On a binary phylogenetic tree, for each site, our revised FastML then works as follows:

1. If leaf *i *has character *b*, then, for each *a *∈ *S'*, set *C*_*i*_(*a*) = *b *and *L*_*i*_(*a*) = .

2. If *i *is an internal node and not the root, its children are *j *and *k*, and it has not yet been processed, then

• if *i *has character *x*, for each *a *∈ *S'*, set *L*_*i*_(*a*) = ·*L*_*j*_(*x*). *L*_*k*_(*x*) and *C*_*i*_(*a*) = *x*;

• otherwise, for each *a *∈ *S'*, set *L*_*i*_(*a*) = max_*c*∈{0,1} _·*L*_*j*_(*c*)·*L*_*k*_(*c*) and *C*_*i*_(*a*) = argmax_*c*∈{0,1} _·*L*_*j*_(*c*)·*L*_*k*_(*c*).

3. If there remain unvisited nonroot nodes, return to Step 2.

4. If *i *is the root node, with children *j *and *k*, assign it the value *a *∈ {0,1} that maximizes π_*a*_·*L*_*j*_(*a*)·*L*_*k*_(*a*), if the character of *i *is not already identified as *x*.

5. Traverse the tree from the root, assigning to each node its character by *C*_*i*_(*a*).

### Refining leaf networks: RefineFast

*RefineFast *uses the parent networks inferred by FastML to evolve new sample leaf networks. Because the strategy is just one of sampling, we do not alter the gene contents of the original leaves--duplication and loss are not taken into account in this refinement step. Let *A*_*l *_and *A*_*p *_be the adjacency matrices of a leaf network and its parent network, respectively, and let  stand for the refined network for *A*_*l*_; then the revised *RefineFast *algorithm carries out the following steps:

1. For each entry(*i*, *j*) of each leaf network *A*_*l*_,

• if *A*_*l*_(*i*, *j*) ≠ *x *and *A*_*p*_(*i*, *j*) ≠ *x*, evolve *A*_*p*_(*i*, *j*) by *P *to get  (*i*, *j*);

• otherwise, assign (*i*, *j*) = *A*_*l*_(*i*, *j*).

2. Use the (*i*, *j*) to replace *A*_*l*_(*i*, *j*).

In this algorithm, the original leaf networks are used only in the first round of ancestral reconstruction, after which they are replaced with the sample networks drawn from the distribution of possible children of the parents.

### Refining leaf networks: RefineML

To make use of the prior information (in the original leaf networks), *RefineML *uses a *belief coefficient k*_*b *_for each entry of the adjacency matrices of these networks, which represents how much we trust the prediction by the base network inference algorithm. With the extended network evolution model, the value of *k*_*b*_is the combination of two items. One is the weights of the edges given by the inference algorithm, which can be calculated from the *conditional probability table *(CPT) parameters of the predicted networks in the DBN framework. The other depends on the distribution of the orthologs of corresponding genes over other leaves. Denote the number of leaves by *N*_*l*_, and the distance between leaf *i *and leaf *j *in the phylogenetic tree by *d*_*ij*_, then the second item of *k*_*b *_of a certain entry for leaf *k *can be calculated by

where *h*_*i *_= 0 if leaf *i *has the corresponding genes, *h*_*i *_= 1 otherwise.

As in *RefineFast*, the refinement procedure does not alter the gene contents of the leaves. Using the same notations as for FastML and *RefineFast*, *RefineML *aims to find the  which maximizes the likelihood of the subtree between *A*_*p*_and . The revised *RefineML *algorithm thus works as follows:

1. Learn the CPT parameters for the leaf networks reconstructed by the base inference algorithm and calculate the *belief coefficient k*_*b *_for every site.

2. For each entry(*i*, *j*) of each leaf network *A*_*l*_, do:

• If *A*_*l*_(*i*, *j*) ≠ *x *and *A*_*p*_(*i*, *j*) ≠ *x*, let *a *= *A*_*p*_(*i*, *j*), *b *= *A*_*l*_(*i*, *j*),

(a) let *Q*(*c*) = *k*_*b *_if *b *= *c*, 1 - *k*_*b *_otherwise;

(b) calculate the likelihood *L*(*a*) = max_*c*∈{0,1} _*p*_*ac*_·*Q*(*c*);

(c) assign (*i*, *j*) = arg max_*c*∈{0,1} _*p*_*ac*_·*Q*(*c*).

• Otherwise, assign (*i*, *j*) = *A*_*l*_(*i*, *j*).

3. Use (*i*, *j*) to replace *A*_*l*_(*i*, *j*).

## Experimental design

To test the performance of our approach, we need regulatory networks as the input to our refinement algorithms. In our simulation experiments, we evolve networks along a given tree from a chosen root network to obtain the "true" leaf networks. Then, in order to reduce the correlation between generation and reconstruction of networks, we use the leaf networks to create simulated expression data and use our preferred network inference method to reconstruct networks from the expression data. These inferred networks are the true starting point of our refinement procedure--we use the simulated gene expression data only to achieve better separation between the generation of networks and their refinement, and also to provide a glimpse of a full analysis pipeline for biological data. We then compare the inferred networks after and before refinement against the "true" networks (generated in the first step).

Despite of the advantages of such simulation experiments (which allow an exact assessment of the performance of the inference and refinement algorithms), results on biological data are highly desirable, as such data may prove quite different from what was generated in our simulations. TFBS data is used to study regulatory networks, assuming that the regulatory interactions determined by transcription factor (TF) binding share many properties with the real interactions [19,20,25]. Given this close relationship between regulatory networks and TFBSs and given the large amount of available data on TFBSs, we chose to use TFBS data to derive regulatory networks for the organisms as their "true" networks--rather than generate these networks through simulation. In this fashion, we produce datasets for the *cis*-regulatory modules (CRMs) for 12 species of *Drosophila*.

With the extended evolutionary model, conducting experiments with real data involves several extra steps besides the refinement step, each of which is a potential source of errors. For example, assuming we have identified gene families of interest, we need to build gene trees or assign orthologies for these genes to be able to reconstruct a history of duplications and losses. Any error in gene tree reconstruction or orthology determination leads to magnified errors in the history of duplications and losses. Assessing the results under such circumstances (no knowledge of the true networks and many complex sources of error) is not possible, so we turned to simulation for this part of the testing. This decision does not prejudice our ability to apply our approach to real data and to infer high-quality networks: it only reflects our inability to compute precise accuracy scores on biological data.

### Experiments on biological data with the basic evolutionary model

We use regulatory networks derived from TFBS data as the "true" networks for the organisms rather than generating these networks through simulations. Such data is available for the *Drosophila *family (whose phylogeny is well studied) with 12 organisms: *D. simulans*, *D. sechellia*, *D. melanogaster*, *D. yakuba*, *D. erecta*, *D. ananassae*, *D. pseudoobscura*, *D. persimilis*, *D. willistoni*, *D. mojavensis*, *D. virilis*, and *D. grimshawi*. The TFBS data is drawn from the work of Kim *et al*. [22], where the TFBSs are annotated for all 12 organisms on 51 CRMs.

We conduct separate experiments on different CRMs. For each CRM, we choose orthologous TFBS sequences of 6 transcription factors (TFs): *Dstat*, *Bicoid*, *Caudal*, *Hunchback*, *Kruppel*, and *Tailless*, across the 12 organisms. Then for each organism, we can get a network with these 6 TFs and the target genes indicated by the TFBS sequences, where the arcs are determined by the annotation of TFBSs and the weights of arcs are calculated from the binding scores provided in [22]. (In this paper we do not distinguish TFs and target genes and call them all "genes.") These networks are regarded as the "true" regulatory networks for the organisms.

Gene-expression data is then generated from these "true" regulatory networks; data is generated independently for each organism, using procedure *DBNSim*, based on the DBN model [11]. Following [14], *DBNSim *uses binary gene-expression levels, where 1 and 0 indicate that the gene is, respectively, *on *and *off*. Denote the expression level of gene *g*_*i *_by *x*_*i*_, *x*_*i *_∈ {0,1}; if *m*_*i *_nodes have arcs directed to *g*_*i *_in the network, let the expression levels of these nodes be denoted by the vector *y *= *y*_1_*y*_2 _⋯  and the weights of their arcs by the vector *w *= *w*_1 _= *w*_2 _⋯ . From *y *and *w*, we can get the conditional probability *Pr*(*x*_*i*_|*y*). Once we have the full parameters of the leaf networks, we generate simulated time-series gene-expression data. At the initial time point, the value of *x*_*i *_is generated by the initial distribution *Pr*(*x*_*i*_); *x*_*i *_at time *t *is generated based on *y *at time *t *- 1 and the conditional probability *P*_*r*_(*x*_*i*_|*y*). We generate 100 time points of gene-expression data for each network in this manner. With this data we can apply our approach. *DBI *is applied to infer regulatory networks from the gene-expression data. The inferred networks are then refined by *RefineFast *and *RefineML*. The whole procedure is run 10 times to provide smoothing and we report average performance over these runs.

### Experiments on simulated data with the extended model

#### Data simulation

In these experiments, the "true" networks for the organisms and their gene-expression data are both generated, starting from three pieces of input information: the phylogenetic tree, the network at the root, and the evolutionary model. While simulated data allows us to get absolute evaluation of our refinement algorithms, specific precautions need to be taken against systematic bias during data simulation and result analysis. We use a wide variety of phylogenetic trees from the literature (of modest sizes: between 20 and 60 taxa) and several choices of root networks, the latter variations on part of the *yeast *network from the KEGG database [26], as also used by Kim *et al*. [3]; we also explore a wide range of evolutionary rates, especially different rates of gene duplication and loss. The root network is of modest size, between 14 and 17 genes, a relatively easy case for inference algorithms and thus also a more challenging case for a boosting algorithm.

We first generate the leaf networks that are used as the "true" regulatory networks for the chosen organisms. Since we need quantitative relationships in the networks in order to generate gene-expression data from each network, in the data generation process, we use adjacency matrices with signed weights. Weight values are assigned to the root network, yielding a weighted adjacency matrix *A*_*p*_. To get the adjacency matrix for its child *A*_*c*_, according to the extended network evolution model, we follow two steps: evolve the gene contents and evolve the regulatory connections. First, genes are duplicated or lost by *p*_*d*_and *p*_*l*_. If a duplication happens, a row and column for this new copy will be added to *A*_*p*_, the values initialized either according to the *neutral initialization *model or the *inheritance initialization *model. (We conducted experiments under both models.) We denote the current adjacency matrix as . Secondly, edges in  are mutated according to *p*_01 _and *p*_10 _to get *A*_*c*_. We repeat this process as we traverse down the tree to obtain weighted adjacency matrices at the leaves, which is standard practice in the study of phylogenetic reconstruction [27,28].

To test our refinement algorithms on different kinds of data, besides *DBNSim*, we also use Yu's GeneSim[29] to generate continuous gene-expression data from the weighted leaf networks. Denoting the gene-expression levels of the genes at time *t *by the vector *x*(*t*), the values at time *t *+ 1 are calculated according to *x*(*t *+ 1) = *x*(*t*) + (*x*(*t*) - *z*)*C *+ ε, where *C *is the weighted adjacency matrix of the network, the vector *z *represents *constitutive expression values *for each gene, and ε models noise in the data. The values of *x*(0) and *x*_*i*_(*t*) for those genes without parents are chosen uniformly at random from the range [0,100], while the values of *z *are all set to 50. The term (*x*(*t*) - *z*)*C *represents the effect of the regulators on the genes; this term needs to be amplified for the use of *DBI*, because of the required discretization. We use a factor *k*_*e *_with the regulation term (set to 7 in our experiments), yielding the new equation *x*(*t *+ 1) = *x*(*t*) + *k*_*e *_(*x*(*t*)- *z*)*C *+ ε.

#### Groups of experiments

With two data generation methods, *DBNSim *and GeneSim, and two base inference algorithms, *DBI *and *DEI*, we can conduct experiments with different combinations of data generation methods and inference algorithms to verify that our boosting algorithms work under all circumstances. First, we use *DBNSim *to generate data for *DBI*. 13 × *n *time points are generated for a network with *n *genes, since larger networks generally need more samples to gain inference accuracy comparable to smaller ones. Second, we apply *DEI *to datasets generated by GeneSim to infer the networks. Since the *DEI *tool TRNinfer does not accept large datasets (with many time points), here we use smaller datasets than the previous group of experiments with at most 75 time points. For each setup, experiments with different rates of gene duplication and loss are conducted.

For each combination of rates of gene duplication and loss, data generation methods, and base network inference methods, we get the networks inferred by *DBI *or *DEI *for the family of organisms. We then run refinement algorithms on each set of networks with different gene duplication and loss histories: the *duplication-only *history, the *loss-only *history, the history reconstructed by FastML given the true orthology assignment, and that reconstructed by Notung [17] without orthology information as input. Besides, since simulation experiments allow us to record the true gene duplication and loss history during data generation, we can also test the accuracy of the refinement algorithms with the true history, without mixing their performance with that of gene tree reconstruction or reconciliation. Each experiment is run 10 times to obtain average performance.

### Measurements

We want to examine the predicted networks at different levels of sensitivity and specificity. For *DBI*, on each dataset, we apply different penalty coefficients to predict regulatory networks, from 0 to 0.5, with an interval of 0.05, which results in 11 discrete coefficients. For each penalty coefficient, we apply *RefineFast *and *RefineML *on the predicted networks. For *DEI*, we also choose 11 thresholds for each predicted weighted connection matrix to get networks on various sparseness levels. For each threshold, we apply *RefineFast *on the predicted networks. We measure specificity and sensitivity to evaluate the performance of the algorithms and plot the values, as measured on the results for various penalty coefficients (for *DBI*) and thresholds (for *DEI*) to yield ROC curves. In such plots, the larger the area under the curve, the better the results.

## Results and analysis

### Results on biological data with the basic evolutionary model

**Figure 1:**
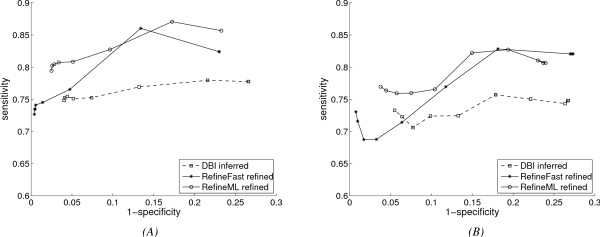
**Performance of refinement algorithms on *Drosophila *data, with basic network evolution model**. (A) Results on CRM abd-A_iab-2_1.7_; (B) Results on CRM Abd-B_IAB5.

We conducted experiments on different CRMs of the 12 *Drosophila *species; here we show results on two of them. In both experiments, regulatory networks have 6 TFs and 12 target genes, forming networks with 18 nodes. Average performance for the base inference algorithm (*DBI*) and for the two refinement algorithms over 10 runs for these two experiments is shown in Fig. 1 using ROC curves. In the two plots, the points on each curve are obtained with different structure complexity penalty coefficients. From Fig. 1 we can see the improvement of our refinement algorithms over the base algorithm is significant: *RefineML *improves significantly both sensitivity and specificity, while *RefineFast *loses a little sensitivity while gaining more specificity for sparse networks. (In both CRMs, the standard deviation on sensitivity is around 0.05 and that on specificity around 0.005.) The dominance of *RefineML *over *RefineFast *shows the advantage of reusing the leaf networks inferred by base algorithms, especially when the error rate in these leaf networks is low. Results on other CRMs show similar improvement of our refinement algorithms. Besides the obvious improvement, we can also observe the fluctuation of the curves: theoretically sensitivity can be traded for specificity and vice versa, so that the ROC curves should be in "smooth" shapes, which is not the case for Fig. 1. Various factors can account for this: the shortage of gene-expression data to infer the network, the noise inherent in biological data, the special structure of the networks to be inferred, or the relatively small amount of data involved, leading to higher variability. (We have excluded the first possibility by generating larger gene-expression datasets for inference algorithms, where similar fluctuations still occur.)

We analyze the difference level between the "true" networks of the 12 organisms, to obtain a view of the evolutionary rate of regulatory networks in our datasets. For each CRM, we take the union of the edges in all 12 networks, classify these edges by the number of networks in which they are present, and calculate the proportion of each category. The overall proportions on all CRMs are shown in Table 1; a large fraction of edges are shared by less than half of the organisms, meaning that the networks are quite diverse. Therefore, the improvement brought by the refinement algorithms is due to the use of phylogenetic information rather than the averaging effect of trees.

**Table 1:** The proportion of edges shared by different numbers of species

Number of species	1	2	3	4	5	6	7	8	9	10	11	12
Proportion of edges	0.19	0.18	0.03	0.07	0.03	0.09	0.03	0.09	0.02	0.07	0.07	0.13
Cumulative fraction	0.19	0.37	0.40	0.47	0.50	0.59	0.62	0.71	0.73	0.80	0.87	1.00

### Results with extended evolutionary model

We used different evolutionary rates to generate the networks for the simulation experiments. In [11] we tested mainly edge gain or loss rates; here we focus on testing different gene duplication and loss rates. We also conducted experiments on various combinations of gene-expression data generation methods and network inference methods. The inferred networks were then refined by refinement algorithms with different models of gene duplications and losses. We do not directly compare the extended model with the basic, as the two do not lend themselves to a fair comparison -- for instance, the basic model requires equal gene contents across all leaves, something that can only be achieved by restricting the data to a common intersection, thereby catastrophically reducing sensitivity.

Since the results of using *neutral initialization *and *inheritance initialization *in data generation are very similar, we only show results with the *neutral initialization *model. We first refine networks with the true gene duplication and loss history to test the pure performance of the refinement algorithms, then we present and discuss the results of refinement algorithms with several other gene evolution histories, which are more suitable for the application on real biological data. All results we show below are averages over 10 runs.

#### Refine with true history of gene duplications and losses

**Figure 2:**
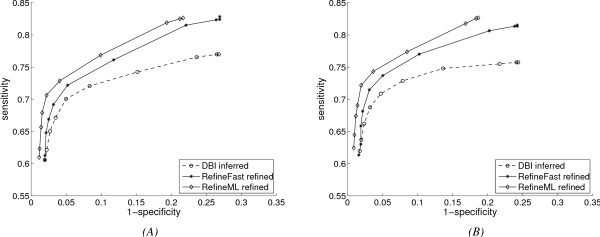
**Performance with extended evolution model and *DBI *inference method, and true history of gene duplications and losses**. (A) Results with higher gene duplication and loss rates; (B) Results with lower gene duplication and loss rates.

In Fig. 2, we show the results of the experiments with *DBNSim *used to generate gene-expression data, and *DBI *as base inference algorithm. All results with *DBI *inference that we show are on one representative phylogenetic tree with 35 nodes on 7 levels, and the root network has 15 genes. The left plot has a relatively high rate of gene duplication and loss (resulting in 20 duplications and 23 losses along the tree), while the right one has a slightly lower rate (with 19 duplications and 15 losses), again averaged over 10 runs.

**Figure 3:**
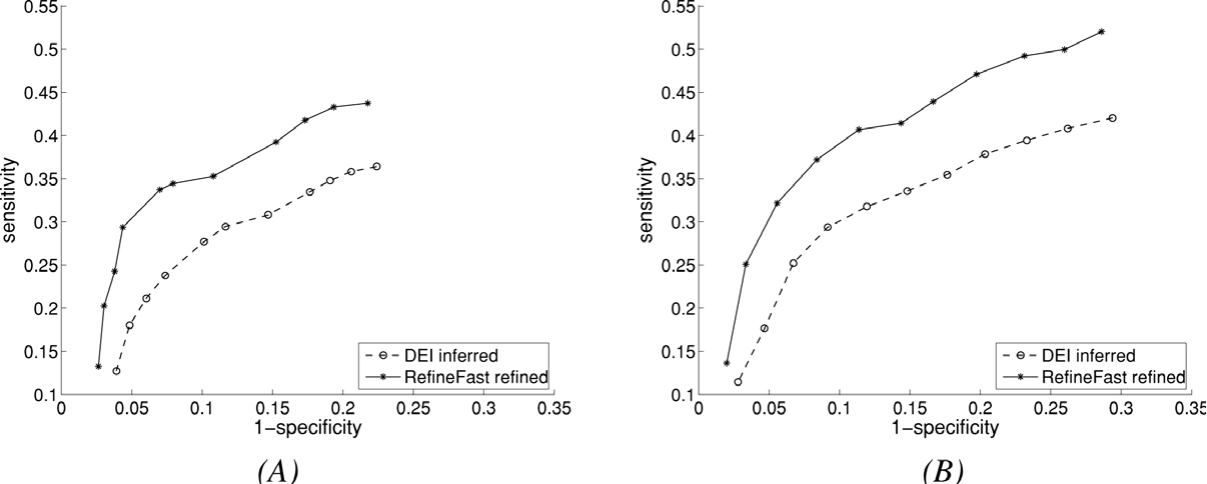
**Performance with extended evolution model and *DEI *inference method, and true history of gene duplications and losses**. (A) Results with higher gene duplication and loss rates; (B) Results with lower gene duplication and loss rates.

Given the size of the tree and the root network, these are high rates of gene duplication and loss, yet, as we can see from Fig. 2, the improvement gained by our refinement algorithms remains clear in both plots, while *RefineML *further dominates *RefineFast *in both sensitivity and specificity, thanks to the appropriate reuse of the inferred leaf networks. In the experiments with *DEI *network inference, GeneSim is used to generate continuous gene-expression data. In these experiments, the root network has 14 genes, and the phylogenetic tree has 37 nodes on 7 levels. The average performance of *DEI *and *RefineFast *over 10 runs is shown in Fig. 3. We also show results for two different evolutionary rates: Fig. 3(A) has higher gene duplication and loss rates, resulting in 15 duplications and 7 losses, while datasets in Fig. 3(B) have an average of 8 duplications and 3 losses. The *DEI *tool aims to infer networks with small gene-expression datasets. *RefineFast *significantly improves the performance of the base algorithm, especially the sensitivity. (Sensitivity for *DEI *is poor in these experiments, because of the inherent lower sensitivity of TRNinfer, as seen in [11] and also because of the reduced size of the gene-expression datasets.) Since the difference between the gene duplication and loss rates in Fig. 3(A) and Fig. 3(B) is large, we can observe more improvement in Fig. 3(B), which has lower rates. This is because high duplication and loss rates give rise to a large overall gene population, yet many of them exist only in a few leaves, so that there is not much phylogenetic information to be used to correct the prediction of the connections for these genes.

#### Refine with *duplication-only *and *loss-only *histories

We have seen from Figs. 2 and 3 that our two refinement algorithms improve the networks inferred by both *DBI *and *DEI*. Since the accuracy of *DBI *is much better than that of *DEI*, which causes more difficulty for refinement algorithms, and since *RefineML *does clearly better than *RefineFast*, hereafter we only show results with *DBI *inference and *RefineFast *refinement, which are on the same datasets as used in Fig. 2.

**Figure 4:**
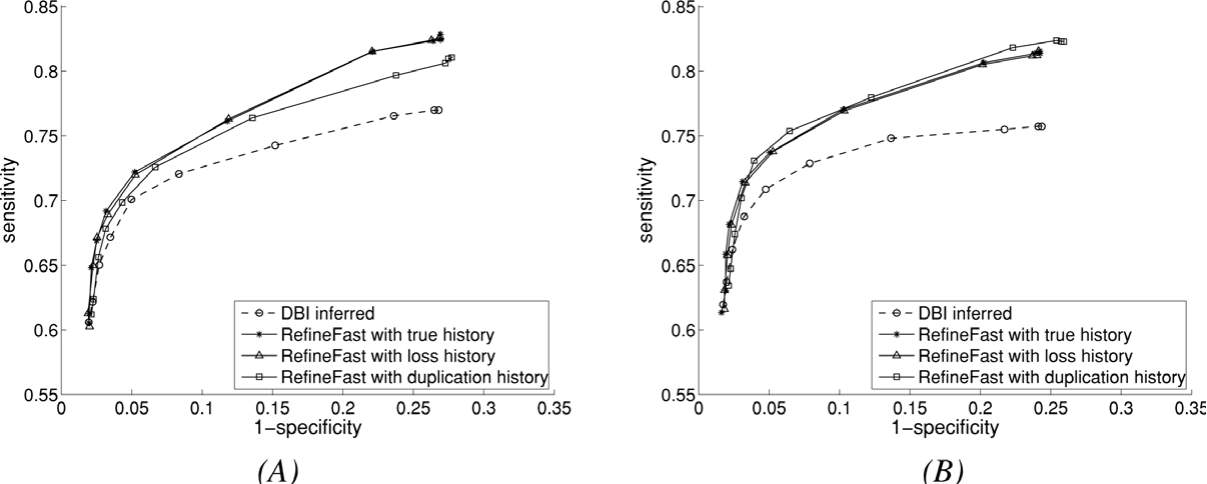
**Performance with extended evolution model and *DBI *inference method, with *duplication-only *and *loss-only *histories**. (A) Results with higher gene duplication and loss rates; (B) Results with lower gene duplication and loss rates.

Fig. 4 shows the comparison of the performance of *DBI *and *RefineFast *with respectively the true gene duplication and loss history, the *duplication-only *history and the *loss-only *history assuming correct orthology assignment. The *duplication-only *and *loss-only *assumptions are at the opposite (and equally unrealistic) extremes of possible models of gene family evolution -- their only positive attribute is that they facilitate the reconstruction of that evolution. Yet we see that *RefineFast *still improves the base network inference algorithm with both models. The performance of the *duplication-only *history differs between Fig. 4(A) and Fig. 4(B): in Fig. 4(A), it does worse than the true history and the *loss-only *history, while in Fig. 4(B), its performance is comparable with the other two. This is because there are more gene losses than gene duplications in the left plot, but more gene duplications than gene losses in the right plot, which the *duplication-only *history matches better. The performance of the *loss-only *history appears to be steady and not much affected by different evolutionary rates.

#### Refine with inferred histories of gene duplications and losses

**Figure 5:**
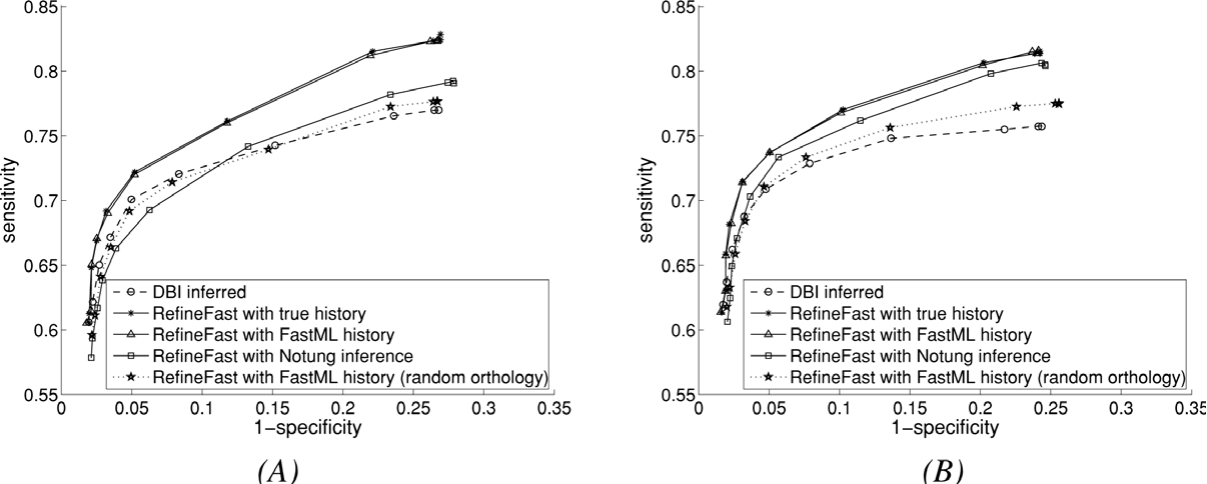
**Performance with extended evolution model and *DBI *inference method, with inferred mixture histories**. (A) Results with higher gene duplication and loss rates; (B) Results with lower gene duplication and loss rates.

In Fig. 5, we show the performance of refinement algorithm with various inferred gene duplication and loss histories, compared to that with the true history. FastML is applied to infer history with correct orthology information as described earlier. To test the value of having good orthology information, we also assign orthologies at random and then use FastML to infer ancestral gene contents. In each run, the refinement procedure with this history is repeated 20 times to get average results over 20 random orthology assignments. Finally, we use Notung to reconstruct a gene duplication and loss history without orthology input; Notung not only infers the gene contents for ancestral networks, but also alters the gene contents of the leaves.

In both Fig. 5(A) and Fig. 5(B) the FastML reconstructed history with correct orthology does as well as the true history. In fact, the history is very accurately reconstructed, which explains why the two curves agree so much. However, with the history reconstructed by FastML under random orthology assignments, the refinement algorithm only improves slightly over the base algorithm. With Notung inference *RefineFast *still dominates *DBI *in Fig. 5(B), but not in Fig. 5(A) which has higher evolutionary rates.

#### On using histories of gene duplications and losses, and orthology assignments

Our experiments with various evolutionary histories lead to several conclusions:

1. Good orthology assignments are important.

2. When we have good orthology assignments, the refinement algorithms need not rely on the true history of gene duplications and losses. We can use the *loss-only *history or the history reconstructed by FastML, both of which are easy to build and lead to performance similar to that of the true history.

## Conclusions and future work

We presented a model, associated algorithms, and experimental results to test the hypothesis that a more refined model of transcriptional regulatory network evolution would support additional refinements in accuracy. Specifically, we presented a new version of our evolutionary approach to refine the accuracy of transcriptional regulatory networks for phylogenetically related organisms, based on an extended network evolution model, which takes into account gene duplication and loss. As these events are thought to play a crucial role in evolving new functions and interactions [8,9], integrating them into the model both extends the range of applicability of our refinement algorithms and enhances their accuracy. Furthermore, to give a comprehensive analysis of the factors which affect the performance of the refinement algorithms, we conducted experiments with different histories of gene duplications and losses, and different orthology assignments. Results of experiments under various settings show the effectiveness of our refinement algorithms with the new model throughout a broad range of gene duplications and losses.

We also collected regulatory networks from the TFBS data of 12 *Drosophila *species and applied our approach (using the basic model), with very encouraging results. These results confirm that phylogenetic relationships carry over to the regulatory networks of a family of organisms and can be used to improve the network inference and to help with further analysis of regulatory systems and their dynamics and evolution.

Our positive results with the extended network evolution model show that refined models can be used in inference to good effect. Our current model can itself be refined by using the widely studied evolution of TFBS [20,22].

## Competing interests

The authors declare that they have no competing interests.

## Authors' contributions

XZ designed, implemented, and ran all simulation and refinement methods; XZ and BMEM collaborated closely in the elaboration of the project, the analysis of the data, and the writing of the manuscript.
